# DBPF-net: dual-branch structural feature extraction reinforcement network for ocular surface disease image classification

**DOI:** 10.3389/fmed.2023.1309097

**Published:** 2024-01-04

**Authors:** Cheng Wan, Yulong Mao, Wenqun Xi, Zhe Zhang, Jiantao Wang, Weihua Yang

**Affiliations:** ^1^College of Electronic Information Engineering, Nanjing University of Aeronautics and Astronautics, Nanjing, China; ^2^Shenzhen Eye Institute, Shenzhen Eye Hospital, Jinan University, Shenzhen, China

**Keywords:** subconjunctival hemorrhage, pterygium, visual recognition, deep learning, computer aided diagnosis

## Abstract

Pterygium and subconjunctival hemorrhage are two common types of ocular surface diseases that can cause distress and anxiety in patients. In this study, 2855 ocular surface images were collected in four categories: normal ocular surface, subconjunctival hemorrhage, pterygium to be observed, and pterygium requiring surgery. We propose a diagnostic classification model for ocular surface diseases, dual-branch network reinforced by PFM block (DBPF-Net), which adopts the conformer model with two-branch architectural properties as the backbone of a four-way classification model for ocular surface diseases. In addition, we propose a block composed of a patch merging layer and a FReLU layer (PFM block) for extracting spatial structure features to further strengthen the feature extraction capability of the model. In practice, only the ocular surface images need to be input into the model to discriminate automatically between the disease categories. We also trained the VGG16, ResNet50, EfficientNetB7, and Conformer models, and evaluated and analyzed the results of all models on the test set. The main evaluation indicators were sensitivity, specificity, F1-score, area under the receiver operating characteristics curve (AUC), kappa coefficient, and accuracy. The accuracy and kappa coefficient of the proposed diagnostic model in several experiments were averaged at 0.9789 and 0.9681, respectively. The sensitivity, specificity, F1-score, and AUC were, respectively, 0.9723, 0.9836, 0.9688, and 0.9869 for diagnosing pterygium to be observed, and, respectively, 0.9210, 0.9905, 0.9292, and 0.9776 for diagnosing pterygium requiring surgery. The proposed method has high clinical reference value for recognizing these four types of ocular surface images.

## 1 Introduction

Pterygium is a common ocular surface disease caused by overgrowth of fibro vascularity in the subconjunctival tissue, resulting in invasion of the inner eyelid and outer cornea (1). It is most prevalent in areas with high ultraviolet light; in some areas, 9.5% of the pterygium patient population is associated with prolonged exposure to high ultraviolet light (2). Clinically, pterygium can be categorized into active and fixed stages. In the fixed stage, the pterygium invades the cornea to a lesser extent, with thin fibro vascular tissue and a smooth, transparent cornea. In the active stage, the pterygium severely invades the cornea, resulting in a cloudy cornea, which if not properly controlled can obscure the pupil and cause irritation and astigmatism, with a more serious effect on vision and limited eye movement accompanied by pain (3). In the medical field, the width of the pterygium (WP) invading the cornea is commonly used as an indicator of whether to operate; the patient is in the stage to be observed when the width of invasion is less than 3 mm, and in the stage to be operated when the width of invasion is greater than 3 mm (4). Subconjunctival hemorrhage is also a common ocular surface disease characterized by painless, acute, obvious red, swollen hemorrhages in the absence of secretions under the conjunctiva, which may evolve from punctate to massive hemorrhages, rendering the underlying sclera invisible (5, 6). Subconjunctival hemorrhage can be defined histologically as bleeding between the conjunctiva and the outer layer of the sclera, and the blood component will be found in the lamina propria of the conjunctiva when the blood vessels under the conjunctiva rupture (7). In contrast to pterygium, subconjunctival hemorrhage is not vision-threatening and is predominantly found in hypertensive groups over 50 years of age (8). Pterygium and subconjunctival hemorrhage often cause uneasiness and anxiety in patients; however, most cases do not require much medical management in the early stages.

Traditional screening methods for ocular surface diseases rely primarily on capturing anterior segment images using a slit lamp for patient sampling, followed by clinical diagnosis by experienced ophthalmologists for early screening and analysis. However, a lack of ophthalmologists in remote areas with poor healthcare resources means that screening for ocular surface diseases still faces great difficulties.

Recently, the increasing application of artificial intelligence in ophthalmology has led to the rapid development of research on intelligent ophthalmic diagnosis. Many researchers have used deep learning algorithms to detect common fundus diseases on fundus images (9–13). In addition, researchers have used deep learning for the diagnosis of ocular surface diseases. In 2018, Zhang et al. implemented an interpretable and scalable deep learning automated diagnostic architecture for four ophthalmic diseases, including subconjunctival hemorrhage and pterygium (14). In 2020, a team from the U.S. improved VggNet16 and applied transfer learning to apply it to screening for pterygium (15). In 2022, Wan et al. improved the U-Net++ segmentation algorithm and proposed a system to diagnose and measure the progression of pterygium pathology (16). To provide high-quality diagnostic services for ocular surface diseases, we designed an automatic diagnostic model for ocular surface diseases using deep learning techniques. The proposed model simultaneously accomplishes the detection of multiple diseases from ocular surface images and achieves fast recognition with high accuracy. This capability is crucial for early screening of ocular surface diseases in remote areas where access to professional medical personnel and equipment is limited.

## 2 Dataset description

The dataset used in this study was provided by the Affiliated Eye Hospital of Nanjing Medical University, and contains color images of the ocular surface with good image quality captured by a professional ophthalmologist. To prevent the leakage of patients’ personal information, the images do not contain patients’ personal information, including but not limited to age, sex, and name.

**FIGURE 1:**
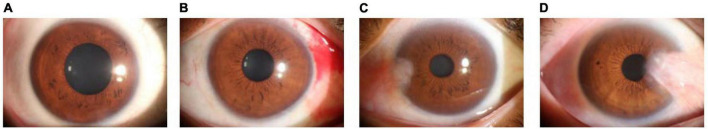
Examples of ocular surface samples. **(A)** Normal ocular surface; **(B)** subconjunctival hemorrhages; **(C)** pterygium to be observed; and **(D)** pterygium requiring surgery.

In this study, 2855 ocular surface images were collected from patients of different age groups and sexes, including 1312 normal ocular surfaces, 251 ocular surface hemorrhages, 909 pterygiums to be observed, and 383 pterygiums requiring surgery. Examples of the four types of ocular surface images are shown in Figure 1. The camera used was a Canon DSLR, model Canon EOS 600D, with diffuse illumination from a slit lamp and an image resolution of 5184 × 3456. The quality of the images was verified by a professional ophthalmologist. We followed the guidelines proposed by Yang et al. (17).

## 3 Materials and methods

Currently, image classification algorithms based on deep learning are primarily composed of convolutional neural networks or visual transformer modules. Convolutional neural networks were first proposed by Lecun et al. (18), and several representative modeling algorithms have subsequently emerged. Among them, the residual network architecture proposed by He et al. (19) is an important milestone in the field of computer vision that solves the problem of network training difficulty owing to gradient vanishing and gradient explosion in convolutional neural networks. The vision transformer (20), proposed by researchers at Google Brain, is an image classification algorithm based on the transformer model that allows images to be viewed as sequences and uses a self-attention mechanism to extract features. Traditional convolutional neural networks perform excellently in the field of image processing; however, the convolutional kernel limits its receptive field and may ignore global information in the image. The transformer can consider all the pixels in the image simultaneously, thus capturing global information more reliably. We adopted the conformer model as the main body, which combines the convolutional neural network and transformer models by parallel fusion to fuse local and global features effectively (21). In addition, we propose a structural feature extraction block composed of a patch merging layer and a FReLU layer (PFM block), which improves the conformer to further differentiate between pterygium to be observed and pterygium to be operated. We propose this dual-branch network reinforced by PFM block (DBPF-Net).

### 3.1 Network structure

In computer vision, local and global features are an important pair of concepts that have been extensively studied in the long history of visual feature description. Local features characterize local regions of images and are represented by compact vectors in local image domains (22); global features include contour representations, shape descriptors, and object representations at long distances (23). Local features provide information about the details in the image, whereas global features provide information about the image as a whole. Using both local and global features helps improve the model performance. In deep learning, a convolutional neural network collects local features in a hierarchical manner through convolutional operations and retains local cues as feature maps, and a vision transformer aggregates global representations in compressed plots by cascading self-attention modules. The conformer efficiently fuses local and global features through concatenation and bridging.

**FIGURE 2:**
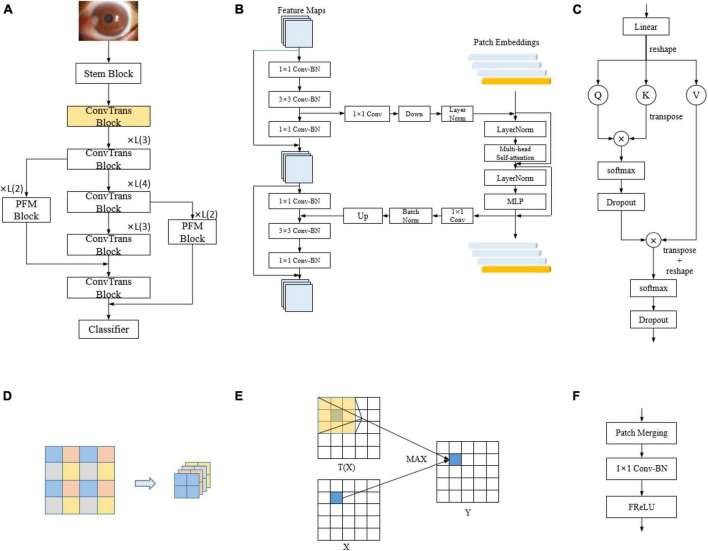
Model structure. **(A)** DBPF-net; **(B)** ConvTrans block; **(C)** multi-head self-attention; **(D)** schematic of patch merging; **(E)** schematic of the FReLU activation function, which can be expressed as **Y**=**M***AX*(**X**,**T**(**x**)); and **(F)** PFM block.

The overall architecture of the conformer is shown as the backbone in Figure 2A, which is mainly composed of ConvTrans blocks. The stem block, consisting of a 7 × 7 convolutional layer with stride 2 followed by a 3 × 3 max pooling layer with stride 2, is used to extract the initial local features, which are then fed into the two branches. The internal structure of the ConvTrans block is shown in Figure 2B. This consists of the convolutional neural network (CNN) branch on the left and the transformer branch on the right, with the feature interactions between them accomplished by the upsampling and downsampling branches. The local features extracted from the CNN branch are transformed into the form of patch embeddings for the transformer branch through the downsampling branch; the global features extracted from the transformer branch are transformed into the form of feature maps for the CNN branch through the upsampling branch. The downsampling operation of the downsampling branch is performed through the max pooling layer, whereas the upsampling operation of the upsampling branch is performed through bilinear interpolation. In every ConvTrans block except for the first one, there are upsampling and downsampling branches for feature exchange.

The core of the transformer branch is multi-head self-attention (24), as shown in Figure 2C. Multi-head self-attention is a technique that introduces multiple heads into the self-attention mechanism, which is used to process sequential data and assign a weight to each element in the sequence to better capture the relationships between them. In the traditional self-attention mechanism, only one head is used to compute the attention weights. In contrast, the multi-head self-attention mechanism introduces multiple heads, each of which has its own weight calculation system to learn different semantic information, thus improving the expressive power of the model.

The input sequence X is first subjected to three different linear transformations to obtain the representations of Q (query), K (key), and V (value). Subsequently, Q, K, and V are divided into multiple heads, denoted *Q_i_*, *K_i_*, *V_i_*. Then, for each head, the attention weights are computed separately by computing the dot product of *Q_i_* and *K_i_* and then performing softmax normalization. Next, a weighted summation is performed on *V_i_* using the attention weights to obtain the attention output for each head, which is concatenated and linearly transformed to obtain the final multi-head self-attention output. The calculation procedure is shown in Eqs 1–4, where $W_{Q}\in R^{d_{model}\times d_{model}}$, $W_{K}\in R^{d_{model}\times d_{model}}$, $W_{V}\in R^{d_{model}\times d_{model}}$, $W_{i}^{Q}\in R^{d_{model}\times d_{k}}$, $W_{i}^{K}\in R^{d_{model}\times d_{k}}$, $W_{i}^{V}\in R^{d_{model}\times d_{v}}$, and $W^{0}\in R^{hd_{v}\times d_{model}}$


(1) $$Q=X\,W_{Q};K=X\,W_{K};V=X$$

(2) $$M\,u\,l\,t\,i\,H\,e\,a\,d\left(Q,K,V\right)=C\,o\,n\,c\,a\,t\left(h\,e\,a\,d_{1},\ldots ,h\,e\,a\,d_{h}\right)\,W^{o}$$

(3) $$h\,e\,a\,d_{i}=A\,t\,t\,e\,n\,t\,i\,o\,n\,\left(Q\,W_{i}^{Q},K\,W_{i}^{K},V\,W_{i}^{V}\right)$$

(4) $$A\,t\,t\,e\,n\,t\,i\,o\,n\left(Q_{i},K_{i},V_{i}\right)=s\,o\,f\,t\,m\,a\,x\,\left(\frac{Q_{i}\,K_{i}^{T}}{\sqrt{d_{k}}}\right)\,V_{i}$$

The PFM block comprises two novel layer structures: the patch merging (25) and flexible rectified linear unit (FReLU) non-linear activation layers (26). The operating principles for these are shown in Figures 2D, E, respectively. Patch merging acts as downsampling for resolution reduction, which is a similar operation to pooling; however, unlike pooling, patch merging does not lose feature information. FReLU is a context-conditional activation function that relies on the local information of the center pixel to obtain pixel-level constructive capabilities. It operates on a localization of the feature map through a parameter-learning convolution kernel, compares it with the center pixel point, and takes the maximum value. This provides each pixel with an option to view the contextual information, which enables spatial structure extraction of the feature map. Formally, the joint action of multiple FReLUs can provide a wider selection of information for each pixel, which helps focus on the structural features of the pterygium and differentiate effectively between the two subclasses of pterygium. The structure of the PFM block is shown in Figure 2F, where the downsampling operation is performed by patch merging, followed by a 1 × 1 convolutional layer to change the number of channels. Finally, non-linear activation is performed by the FReLU activation function, which is in line with the design concept of traditional convolutional neural networks.

We designed the PFM block to further differentiate between the similar cases of pterygium to be observed and pterygium requiring surgery. The relationship between the spatial structure of the pterygium and the cornea is particularly important in the medical field, where the depth of pterygium invasion into the cornea is usually used as a discriminator. Relying on the basic lossless downsampling of patch merging and the spatial structure feature extraction of the FReLU activation function, the bottom-layer feature map output from the first 3 × 3 convolutional layer in the ConvTrans block is passed to the top-layer feature map through the two processes of the PFM block, which causes the network to focus on extracting the spatial structure features. The overall architecture of DBPF-Net is shown in Figure 2A.

### 3.2 Data division and pre-processing

The original dataset used in this study consisted of 2855 ocular surface images. Considering the reliability of the model’s performance on the validation set and its generalization on the test set, we divided the dataset into training, validation, and test sets in a ratio of 7:1:2. In the original dataset, there is generally only one image for an eye, and images from the same eye only appear inside one dataset (i.e., training set, validation set, test set). The number of samples for each category in each subset is shown in Table 1. Owing to the different difficulties in obtaining samples for each image category, the number of samples for the four categories is not balanced, which may lead to the model focusing excessively on categories with a large number of training samples and lack of attention to categories with a small number of samples. Therefore, we used enhancement methods to reduce the impact of data imbalance, including image bilinear interpolation stretching, random horizontal flipping, random small-angle rotation, central region cropping, and normalization. The purpose of these steps was to minimize the influence of the upper and lower eyelids during training while preserving the conjunctiva. These augmentation techniques do not eliminate the pathological regions present in the original images, such as hemorrhages on the conjunctiva and pterygium invading the cornea.

**TABLE 1:** Data division.

Category	Train	Validation	Test	Total
C0	919	131	262	1,312
C1	176	25	50	251
C2	637	91	181	909
C3	269	38	76	383
Total	2001	285	569	2,855

C0, C1, C2, and C3, respectively, represent normal ocular surface, subconjunctival hemorrhages, pterygium to be observed, and pterygium requiring surgery.

### 3.3 Model training

We used the Adam optimization algorithm (27) during model training, with a weight decay of 0.0005. The training batch size was 4, the total number of training iterations was 90, and the initial value of the learning rate was 0.0001. Two cross-entropy loss functions were used to supervise the classifiers of the two branches separately, and the importance of the loss function was identical for both. The learning rate was adjusted dynamically using the cosine annealing strategy (28), which helps prevent the model from falling into local optimal solutions during the training process, as well as to avoid the impact of sudden learning rate changes on the training process. In addition, we selected VGG16 (29), ResNet50 (19), EfficientNetB7 (30), and conformer models to compare the classification results, all of which used ImageNet pretrained model parameters as initial conditions.

The central processor used in our experiments was a 3.6 GHz Intel i7-7700, and the graphics processor was an NVIDIA RTX 2080Ti with 11 GB of RAM. The operating system was Windows 10, the programming language was Python 3.6, and the deep learning framework was Pytorch 1.7.

### 3.4 Model evaluation indicators

This study is a multi-categorization task, and we evaluate the effectiveness of the model from two perspectives. The first approach involves evaluating the overall performance of multi-class classification using the kappa coefficient, which demonstrates consistent agreement. The calculation of the kappa coefficient is based on the confusion matrix, and its value typically ranges from 0 to 1. A higher kappa coefficient indicates a higher level of agreement between the model’s evaluation and the diagnostic assessment by experts. The formula for the kappa coefficient is as follows:


(5) $$k=\frac{p_{o}-p_{e}}{1-p_{e}}$$

(6) $$p_{e}==\frac{a_{1}\times b_{1}+a_{2}\times b_{2}+\ldots +a_{c}\times b_{c}}{n\times n}$$

where *p_o_* is the sum of all correctly classified samples divided by the total number of samples, *a_i_* is the number of true samples in category i, and *b_i_* is the number of predicted samples in category i.

Another approach is to convert a multi-classified problem into multiple independent binary classification problems. For example, to identify the normal ocular surface, the normal ocular surface is labeled as a positive sample, whereas the three categories of subconjunctival hemorrhage, pterygium to be observed, and pterygium requiring surgery are labeled as negative samples. To calculate the evaluation indicators for the binary classification problem, the number of true positive (TP), true negative (TN), false positive (FP), and false negative (FN) samples were first obtained from the confusion matrix, and then the accuracy (ACC), sensitivity (SE), specificity (SP), and F1-score (F1) were calculated. Accuracy indicates the proportion of correctly diagnosed samples to the total number of samples; sensitivity indicates the proportion of samples predicted to be positive and actually positive to the proportion of all actual positive samples; specificity indicates the proportion of samples predicted to be negative and actually negative to the proportion of all actual negative samples; and F1-score is defined as the harmonic average of accuracy and sensitivity, which is meaningful for datasets with unbalanced samples.


(7) $$A\,C\,C=\frac{T\,P+T\,N}{T\,P+F\,N+T\,N+F\,P}$$

(8) $$S\,E=\frac{T\,P}{T\,P+F\,N}$$

(9) $$S\,P=\frac{T\,N}{T\,N+F\,P}$$

(10) $$F\,1=\frac{2\,T\,P}{2\,T\,P+F\,P+F\,N}$$

Receiver operating characteristics (ROC) curves are commonly used to analyze the classification performance of different models, owing to their visualization features. The area under the ROC curve (AUC) was used to evaluate the classification accuracy. Generally speaking, an AUC value of 0.50–0.70 is regarded as a low diagnostic value, 0.70–0.85 is regarded as a general diagnostic value, and 0.85 and above is regarded as a good diagnostic value.

## 4 Results

In this study, 569 ocular surface images were randomly selected as a test set containing 262 images of a normal ocular surface, 50 images of subconjunctival hemorrhage, 181 images of pterygium to be observed, and 76 images of pterygium requiring surgery. The model with the best accuracy on the validation set was considered the optimal model for evaluating the performance of the models on the test set.

**FIGURE 3:**
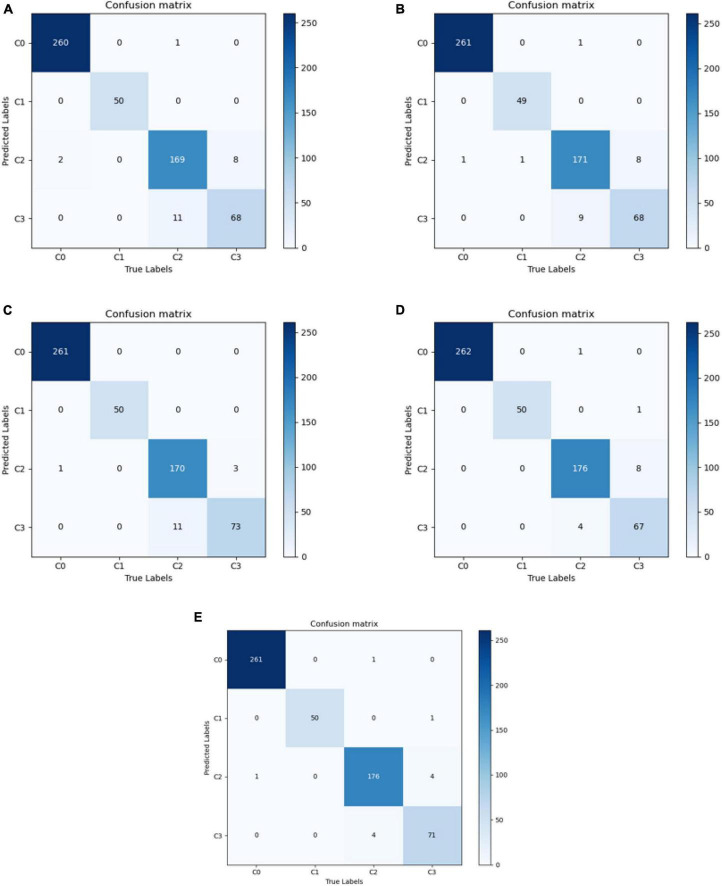
Confusion matrix for each model. **(A)** VGG16; **(B)** ResNet50; **(C)** EfficientNetB7; **(D)** Conformer; and **(E)** DBPF-Net.

The best diagnostic results of each model on the test set are presented in Figure 3, in the form of confusion matrices.

The purpose of this study was to correctly diagnose four categories of ocular surface images: normal ocular surface, subconjunctival hemorrhage, pterygium to be observed and pterygium requiring surgery. To demonstrate the performance of the models clearly, we quantified their evaluation indicators, with results as listed in Table 2. These evaluation indicators are calculated according to Eqs 5–10.

**TABLE 2:** Evaluation indicators for each model in each category.

Models	Evaluation indicators	C0	C1	C2	C3
VGG16	Sensitivity	0.9987 ± 0.0017	0.96 ± 0.0163	0.9152 ± 0.0213	0.8947
	Specificity	0.9945 ± 0.003	1.0	0.9759 ± 0.0012	0.9702 ± 0.0084
	F1-score	0.9962 ± 0.0015	0.9795 ± 0.0084	0.9306 ± 0.0103	0.8577 ± 0.0229
	AUC	1.0	1.0	0.9944 ± 0.0008	0.991 ± 0.0018
	Kappa	0.9319 ± 0.0105			
	Accuracy	0.9548 ± 0.007			
ResNet50	Sensitivity	0.9949 ± 0.0017	1.0	0.9281 ± 0.0078	0.8859 ± 0.0223
	Specificity	0.9967	1.0	0.9742 ± 0.0042	0.9756 ± 0.0028
	F1-score	0.9955 ± 0.0008	1.0	0.9359 ± 0.0021	0.8668 ± 0.0090
	AUC	1.0	1.0	0.9960 ± 0.0001	0.9931 ± 0.0012
	Kappa	0.9389 ± 0.0022			
	Accuracy	0.9595 ± 0.0014			
EfficientNetB7	Sensitivity	0.9962	1.0	0.9355 ± 0.0051	0.9517 ± 0.0123
	Specificity	1.0	1.0	0.9879 ± 0.0024	0.9763 ± 0.0019
	F1-score	0.9981	1.0	0.9539 ± 0.0026	0.9041 ± 0.006
	AUC	0.9999	1.0	0.9909 ± 0.0006	0.9860 ± 0.0009
	Kappa	0.9568 ± 0.0024			
	Accuracy	0.9712 ± 0.0016			
Conformer	Sensitivity	0.9962 ± 0.0031	0.9933 ± 0.0094	0.9668 ± 0.0078	0.8903 ± 0.0223
	Specificity	0.9967 ± 0.0026	0.9987 ± 0.0008	0.9776 ± 0.0044	0.9892 ± 0.0038
	F1-score	0.9962 ± 0.0015	0.9901	0.9597 ± 0.0033	0.9082 ± 0.0031
	AUC	0.9999	1.0	0.9958 ± 0.0002	0.9934 ± 0.0006
	Kappa	0.9583 ± 0.0033			
	Accuracy	0.9723 ± 0.0021			
DBPF-Net	Sensitivity	0.9962 ± 0.0031	1.0	0.9723 ± 0.009	0.9210 ± 0.0186
	Specificity	0.9989 ± 0.001	0.9987 ± .0008	0.9836 ± 0.0048	0.9905 ± 0.0034
	F1-score	0.9974 ± 0.0017	0.9934 ± 0.0046	0.9688 ± 0.0025	0.9292 ± 0.0079
	AUC	0.9989 ± 0.0006	1.0	0.9869 ± 0.0109	0.9776 ± 0.0155
	Kappa	0.9681 ± 0.0022			
	Accuracy	0.9789 ± 0.0014			

C0, C1, C2, and C3, respectively, represent normal ocular surface, subconjunctival hemorrhages, pterygium to be observed, and pterygium requiring surgery. The variable was expressed as the mean ± standard deviation.

**FIGURE 4:**
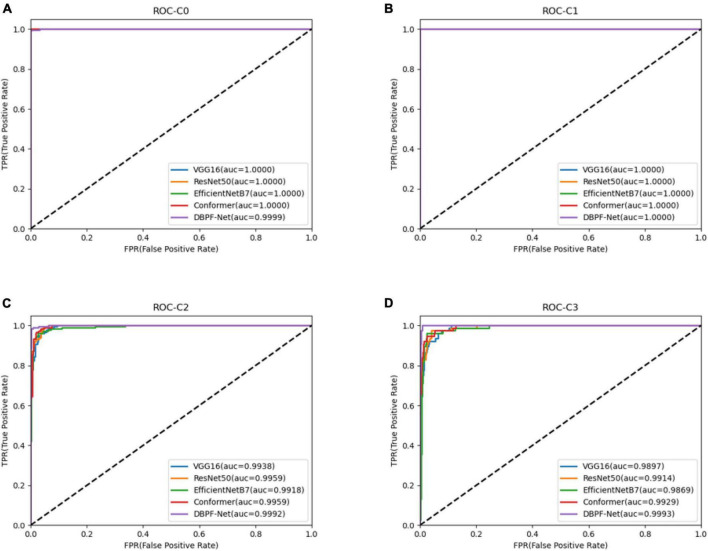
Receiver operating characteristics curves for each model. **(A)** Normal ocular surface; **(B)** subconjunctival hemorrhage; **(C)** pterygium to be observed; and **(D)** pterygium requiring surgery.

**FIGURE 5:**
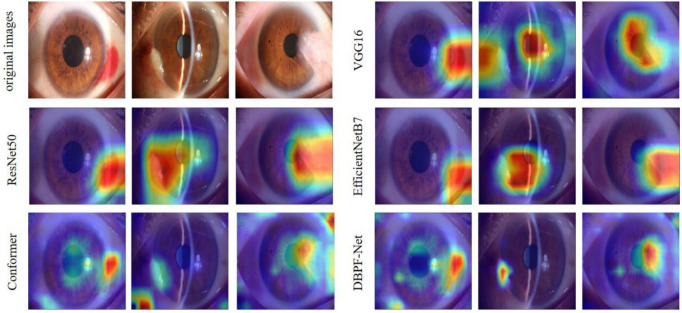
Heat maps of the models for subconjunctival hemorrhage, pterygium to be observed, and pterygium requiring surgery.

In summary, the DBPF-Net model achieved high sensitivity and specificity values, indicating that it performs well in differentiating between positive and negative samples, which is valuable for clinical diagnoses that require accurate identification and differentiation of different disease categories. In addition, its high F1-score and kappa coefficient indicate that the model has excellent classification performance when the data are unbalanced, and high consistency with the evaluation of the expert diagnostic group. The ROC curves for each model with the best accuracy are shown in Figure 4. Moreover, we used Grad-CAM (31) to analyze the region of interest of the models for the ocular surface images, as shown in Figure 5.

## 5 Discussion

Ocular surface diseases have received worldwide attention as a common public health problem. The variety and complexity of these diseases are important factors that should not be ignored in their diagnosis. Therefore, diagnosis and treatment require doctors with rich experience and professional knowledge to be able to determine the condition accurately and take appropriate treatment measures. Currently, the lack of specialized ophthalmologists in areas with a high prevalence of ocular surface diseases leaves many patients without timely diagnosis and treatment. Therefore, it is important to develop an automatic diagnostic model for initial screening and diagnosis.

The application of artificial intelligence to the field of medical image processing has been based on traditional convolutional neural networks and has achieved remarkable research results in recent years. The emergence of vision transformers has confirmed the advantages of global features in image recognition, and a variety of deformation models have been derived (25, 32, 33). The DBPF-Net model proposed in this study selects the conformer as the backbone of the four-way classification model for ocular surface diseases. Compared with other models, the conformer’s ability to extract and fuse global and local features gives it better feature extraction capability. In addition, we propose a PFM block for enhancing the conformer’s extraction of spatial structural features to differentiate further between the two pterygium categories.

Several research groups have investigated the classification and diagnosis of ocular surface diseases. Elsawy et al. (34) employed an improved VGG19 model to classify corneal diseases automatically, achieving an overall F1-score in excess of 86%. Zhang et al. (14) implemented an automated diagnostic architecture with deep learning interpretability and scalability, achieving over 95% accuracy for pterygium. Xu et al. (35) Proposed a computer-aided pterygium diagnosis system based on EfficientNetB6 with transfer learning, achieving a sensitivity of 90.06% for pterygium to be observed and 92.73% for pterygium requiring surgery. Huang et al. (36) developed a deep learning system for pterygium grading, using a classification algorithm to categorize pterygiums from non-pterygiums, and then a segmentation algorithm to segment pterygiums for grading, achieving sensitivities ranging from 80 to 91.67%. These studies exclusively employed CNN models without specific disease-targeted feature modules. Our study focused on the practical situation of whether or not patients with pterygium need surgery. The proposed DBPF-Net achieved an accuracy of 97.89% for the four categories, demonstrating promising results. In our experiments, we compared it with three other representative CNN models and the original conformer model.

As shown in Figure 3 and Table 2, the overall evaluation indicators of DBPF-Net were generally higher than those of the other models. Among the test results for all models, evaluation indicators for the normal ocular surface and subconjunctival hemorrhage categories reached a high level, mainly because of the sufficient number of samples in the normal ocular surface category and the significant characteristics of the subconjunctival hemorrhage category. The test results for the categories of pterygium to be observed and pterygium requiring surgery demonstrate that the conformer model exhibits superior discriminative ability compared to VGG16 and ResNet50, While in comparison with EfficientNetB7, each of the two was dominant. Compared to the conformer model, DBPF-Net showed an improvement of 0.55% in sensitivity, 0.6% in specificity, and 0.91% in F1-score for the category of pterygium to be observed. For the category of pterygium requiring surgery, DBPF-Net achieved an increase of 3.07% in sensitivity and 2.1% in F1-score. Overall, DBPF-Net showed further improvement in pterygium diagnosis compared with Conformer. Although the proposed method has a slightly lower AUC than Conformer, the proposed method outperforms in terms of the F1 Score. The heat map shown in Figure 5 demonstrates that DBPF-Net focuses on the area of hemorrhage in the category of subconjunctival hemorrhage, the area of pterygium tipping into the cornea in the category of pterygium to be observed, and the area of pterygium approaching the center of the cornea in the category of pterygium requiring surgery. The heat maps generated by VGG16, ResNet50, and EfficientNetB7 indicate that their attention on the lesion area is not adequately concentrated, as well as on the pupil area. In comparison, Conformer exhibits a similar focus area to DBPF-Net, the latter is more focused.

Our study has some limitations. First, the dataset used in this study has a limited number of samples and an uneven number of samples per category, which leads to poorer generalization and precision for categories with fewer samples. Second, the hardware configuration of the experimental platform in this study was ordinary, and the model performance was limited by the amount of GPU RAM. In the future we will continue to collect datasets, improve the model to increase its accuracy, and consider a method of semantic segmentation of images to assist in classification.

## 6 Conclusion

In this paper, we propose DBPF-Net, a model that achieves high classification performance on four categories of ocular surface images: normal ocular surface, subconjunctival hemorrhage, pterygium to be observed, and pterygium requiring surgery. This model is hopefully to achieve initial screening for ocular surface diseases in remote areas where access to professional medical personnel and equipment is limited. In addition, we hope to help reduce the workload of medical personnel in primary care facilities.

## Data availability statement

The original contributions presented in the study are included in the article/supplementary material, further inquiries can be directed to the corresponding authors.

## Author contributions

CW: Data curation, Methodology, Software, Writing – original draft. YM: Data curation, Methodology, Software, Writing – original draft. WX: Data curation, Software, Validation, Writing – review and editing. ZZ: Data curation, Validation, Writing – review and editing. JW: Data curation, Formal analysis, Methodology, Project administration, Supervision, Writing – review and editing. WY: Data curation, Formal analysis, Methodology, Project administration, Supervision, Writing – review and editing.
